# Stomatal Development and Conductance of a Tropical Forage Legume Are Regulated by Elevated [CO_2_] Under Moderate Warming

**DOI:** 10.3389/fpls.2019.00609

**Published:** 2019-05-31

**Authors:** Eduardo Habermann, Eduardo A. Dias de Oliveira, Daniele Ribeiro Contin, Juca A. B. San Martin, Lucas Curtarelli, Miquel A. Gonzalez-Meler, Carlos Alberto Martinez

**Affiliations:** ^1^Department of Biology, Faculty of Philosophy, Sciences and Languages of Ribeirão Preto (FFCLRP), University of São Paulo, Ribeirão Preto, Brazil; ^2^Ecology and Evolution, Department of Biological Sciences, University of Illinois, Chicago, IL, United States

**Keywords:** elevated CO_2_, gas exchange, global climate change, stomatal conductance regulation, tropical forage legume, warming

## Abstract

The opening and closing of stomata are controlled by the integration of environmental and endogenous signals. Here, we show the effects of combining elevated atmospheric carbon dioxide concentration (*eCO*_2_; 600 μmol mol^-1^) and warming (+2°C) on stomatal properties and their consequence to plant function in a *Stylosanthes capitata* Vogel (C_3_) tropical pasture. The *eCO*_2_ treatment alone reduced stomatal density, stomatal index, and stomatal conductance (*g_s_*), resulting in reduced transpiration, increased leaf temperature, and leading to maintenance of soil moisture during the growing season. Increased CO_2_ concentration inside leaves stimulated photosynthesis, starch content levels, water use efficiency, and PSII photochemistry. Under warming, plants developed leaves with smaller stomata on both leaf surfaces; however, we did not see effects of warming on stomatal conductance, transpiration, or leaf water status. Warming alone enhanced PSII photochemistry and photosynthesis, and likely starch exports from chloroplasts. Under the combination of warming and *eCO*_2_, leaf temperature was higher than that of leaves from the warming or *eCO*_2_ treatments. Thus, warming counterbalanced the effects of CO_2_ on transpiration and soil water content but not on stomatal functioning, which was independent of temperature treatment. Under warming, and in combination with *eCO*_2_, leaves also produced more carotenoids and a more efficient heat and fluorescence dissipation. Our combined results suggest that control on stomatal opening under *eCO*_2_ was not changed by a warmer environment; however, their combination significantly improved whole-plant functioning.

## Introduction

Since the industrial revolution, anthropogenic activities have increased atmospheric carbon dioxide concentrations ([CO_2_]) from 270 μmol mol^-1^ to the more than 400 μmol mol^-1^ we see today (IPCC, 2018). [CO_2_] is expected to reach 600 μmol mol^-1^ by 2050 as modeled by the Representative Concentration Pathways (RCP; IPCC, 2018). According to the RCP6.0 scenario, elevated [CO_2_] (*eCO*_2_) coupled with the increased emissions of other greenhouses gases (GHG), such as methane (CH_4_) and nitrous oxide (N_2_O), will cause the global mean surface air temperature to rise by 2°C by 2100 if the goals outlined in the Paris Agreement are not met (IPCC, 2018). Therefore, following the predicted changes in climate, studies to understand how the combined changes in CO_2_ and temperature will affect plants and ecosystems are paramount.

There is a considerable body of literature regarding the responses of plants and ecosystems to climate change in temperate systems (Marchi et al., 2004; Ainsworth and Long, 2005; Tubiello et al., 2007; Wall et al., 2011; Rojas-Downing et al., 2017; Urban et al., 2017), including temperate pastures (Lee et al., 2017), which are very important to the sustainability of livestock activities. However, information on the climate change sensitivity of tropical forage species is lacking (Webb et al., 2012; Rojas-Downing et al., 2017; IPCC, 2018), particularly considering that a greater area of livestock pasture is found in the tropics and subtropics. Climate change could affect almost every critical factor related to livestock production (Rojas-Downing et al., 2017) including productivity of forage species (Martinez et al., 2014; Gonzalez-Meler et al., 2017). Because temperature and CO_2_ are important factors affecting plant physiological performance and stomatal control, understanding the impact of **eCO*_2_* and warming on plant performance and stomatal functioning in tropical pastures is vital to understanding the effect of climate change on the productivity of pastures in tropical and subtropical regions (Asner et al., 2004; Tubiello et al., 2007; Prado et al., 2016; Rojas-Downing et al., 2017).

The responses of plants to *eCO*_2_ and warming include complex anatomical, biochemical, and physiological adjustments (Habermann et al., 2019b). Stomatal closure is one of the most common responses of plants to *eCO*_2_ (Morison, 1998). Recently, extensive efforts have been made to understand the underlying mechanisms of CO_2_-sensing in guard-cells and turgor control, particularly under a CO_2_-enriched atmosphere. CO_2_ is a lipophilic molecule that diffuses across membranes and can also move via mass flow through aquaporins (Zhao et al., 2017). In guard cells, CO_2_ activates K^+^_out_ efflux channels that increase the intracellular water potential and result in water efflux and stomatal closure (Brearley et al., 1997; Raschke et al., 2003). However, whether CO_2_-sensing mechanisms are located in guard-cells or mesophyll cells is under debate (Kim et al., 2010). Other effects of *eCO*_2_ on stomatal properties include changes in the differentiation of epidermal cells into stomata, reducing stomatal number, and contributing to decreased *g_s_* (Doheny-Adams et al., 2012).

These responses of stomata to changes in the environment are integrated over changes in stomatal conductance (*g_s_*). Stomatal conductance is sensitive to environmental factors such as light, vapor pressure deficit, boundary layer, and [CO_2_]. An enriched CO_2_-world has the potential to significantly affect whole-plant function by improving water use efficiency (*WUE*; Nadeau and Sack, 2002; Allen et al., 2011) as a consequence of increased net photosynthetic rate and reduced *g_s_*. Reduced *g_s_* leads to a decrease in transpiration rates, a common response of plants to *eCO*_2_, increasing leaf water, leaf temperature and leading to the conservation of soil moisture (Wall et al., 2001; Nelson et al., 2004; Bernacchi et al., 2007; Leakey et al., 2009; Gonzalez-Meler et al., 2017). In contrast, although very uncommon, *g_s_* has been shown to increase in woody plants and plants well-adapted to warm and low moisture conditions in response to increased atmospheric [CO_2_] (Purcell et al., 2018).

In contrast with the effects of *eCO*_2_, warming increases *g*_s_ and leaf transpiration rates (*E*) under adequate conditions of water availability (Chattaraj et al., 2014). Increased temperature stimulates H^+^ efflux through guard cells and increases whole-cell conductance to K^+^, which diffuses to guard cells, promoting water influx (Ilan et al., 1995). This phenomenon may decrease leaf water status (Ruiz-Vera et al., 2013) and *WUE* (Fu et al., 2014; Guan et al., 2016), affecting the ultrastructure of chloroplasts and the functioning of photosystems and enzymes, including the stability of cell membranes. However, in some studies, there was little or no effect of warming on plant water status, as in the case of wheat plants (*Triticum aestivum*, Poaceae) grown at 3/1.5°C (daytime/nighttime) above ambient temperatures (Wall et al., 2011). Depending on the specie’s thermal tolerance, plants can acclimate and warming might stimulate plant photosynthesis (Fu et al., 2014; Martinez et al., 2014).

While the isolated effects of *eCO*_2_ and warming on *g_s_* have been widely investigated, there is little information concerning the combination of these two abiotic factors in tropical environments under field conditions. Imbalances between carbon uptake and hydraulic dynamics under a warmer, CO_2_-enriched atmosphere may affect whole-plant function, causing negative impacts on forage species and livestock production. Most information on stomatal responses are not from open field experiments (Ainsworth and Long, 2005) and to date there are very limited studies on tropical forages in open environments (Martinez et al., 2014; Gonzalez-Meler et al., 2017; Habermann et al., 2019a). Stomatal functioning under field conditions integrates several biotic and abiotic factors that may produce new and unexpected responses. For example, the magnitude of *g_s_* decreases seen under open-air field conditions using FACE technology have been lower than those seen from studies in open-top chambers or growth chambers (Long et al., 2006; Leakey et al., 2009). Also, despite the fact that a multifactorial field approach likely provides more relevant results for predicting plant responses and developing more precise earth systems models (Larsen et al., 2011; Dieleman et al., 2012; Drewniak and Gonzalez-Meler, 2017), studies combining the effects of *eCO*_2_ with warming and other factors are rare (Dieleman et al., 2012).

In this study, we used the Trop-T-FACE facility (Habermann et al., 2019b) that combines a FACE (free-air carbon dioxide enrichment) and a T-FACE (free-air temperature-controlled enhancement) system (Kimball et al., 2008). We aimed to unravel the effects of *eCO*_2_ and warming on stomatal properties and subsequently on plant functioning in *Stylosanthes capitata* Vogel (C_3_). In Brazil, *S. capitata* is one of the most common legume forage species and is often mixed with grasses due to its resistance to disease, high seed yield, and excellent adaptation to acidic and low fertility soils (Miles and Lapointe, 1992; Embrapa, 2014). We tested the hypotheses that (i) under *eCO*_2_ alone, *g_s_*, and stomatal density and index will decrease, conserving soil moisture, decreasing antioxidant defenses, and improving water status and photosynthetic performance; that (ii) under warming alone, *g_s_* and stomatal density will increase, increasing leaf transpiration rates and decreasing soil moisture, but due to the antioxidant response and heat tolerance of *S. capitata*, photosynthesis will increase; and that (iii) under an *eCO*_2_ and warming combination, the effects of *eCO*_2_ on stomatal development and *g_s_* will mitigate the antagonistic effect of warming, leading to enhanced leaf water status, antioxidant defense, and photosynthetic performance.

## Materials and Methods

### Location and Experimental Set-Up

The experiment was conducted during late autumn and early winter of 2015 in the Trop-T-FACE facility (Habermann et al., 2019b; Supplementary Figure 1) located at the University of São Paulo, Ribeirão Preto campus, São Paulo State, Brazil (S 21° 10′ 8″ W 47° 51′ 48.2″). The 2500 m^2^ study area is located 580 m above sea level, and the soil was classified as a dystrophic red Latosol with clay texture. Thirty days before seeding, we performed soil liming to correct the pH of the experimental area from a value of 4.0 to 5.5. We fertilized the soil with NPK 4-14-8 at a dose of 1 ton ha^-1^ 17 days after the soil liming, following the recommendations from Werner et al. (1997). Seeds of *S. capitata* were sown in rows 30 cm apart in 16 plots of 10 m × 10 m. During seedling growth, rainfall was intense and the temperature was high, which stimulated the initial growth of plants (Supplementary Figure 2). Supplemental irrigation was performed when necessary. After approximately three months of vegetative growth and pasture establishment, plants were clipped to 30 cm aboveground, and treatments of *eCO*_2_ and warming were initiated and maintained during the plant reproductive regrowth. Plants reached anthesis in the first week after standardization clipping. Plants were rainfed and maintained under experimental conditions of warming and *eCO*_2_ for 66 days (Supplementary Figure 2A). We chose not to irrigate plants during the experiment to maintain field conditions of soil moisture, a normal practice for this species in the tropics. Warming was provided by the T-FACE system (Kimball et al., 2008) and *eCO*_2_ treatment was applied using the miniFACE system (Miglietta et al., 1999).

We used an experimental design with four randomized blocks to test the effects of two levels of atmospheric [CO_2_]: ambient (*aCO*_2_, current ∼400 μmol mol^-1^) and elevated (*eCO*_2_, ∼600 μmol mol^-1^) and two levels of air temperature: ambient (*aT*) and elevated (*eT*, +2°C above ambient canopy temperature). We combined these two variables in four treatments. Each block contained one plot of each treatment: *aCO*_2_*aT* (ambient [CO_2_] and ambient temperature); *eCO*_2_*aT* (600 μmol mol^-1^ [CO_2_] and ambient temperature); *aCO_2_eT* (ambient [CO_2_] and +2°C above ambient canopy temperature); and *eCO*_2_*eT* (600 μmol mol^-1^ [CO_2_] and +2°C above ambient canopy temperature). The target concentration of 600 μmol mol^-1^ in plots with elevated [CO_2_] was maintained during daylight hours, and the target temperature of 2°C above ambient was maintained 24 h day^-1^ in the heated plots. There were 16 (10 m × 10 m) experimental plots (4 *aCO*_2_*aT* plots, 4 *eCO*_2_*aT* plots, 4 *aCO_2_eT* plots and 4 *eCO*_2_*eT* plots) containing a 2-m diameter experimental ring of CO_2_ fumigation placed at the central portion of the plot. All experimental rings were separated by at least 12 m to avoid CO_2_ cross-contamination (see Supplementary Figure 1).

An automatic microclimate station WS-PH1 connected to a DL2e datalogger (Delta-T Devices Ltd., Cambridge, United Kingdom) was installed in the center of the Trop-T-FACE facility. The station was responsible for monitoring and recording meteorological data: total solar radiation (*Rad*), air temperature (*T_air_*), and relative air humidity (*Rh*) using specific sensors throughout the entire growing season. Precipitation data were obtained from a weather station located near the Trop-T-FACE facility. Volumetric soil water content (*SWC*) and the soil temperature (*T_soil_*) in each experimental ring were measured from 0 to 10 cm deep using 16 ThetaProbe ML2X and ST2 sensors, respectively, both located in the center of each ring. The data were collected and stored on the DL2e datalogger (Delta-T Devices Ltd., Cambridge, United Kingdom).

### Trop-T-FACE Facility

The Trop-T-FACE facility was described by Habermann et al. (2019b) and in short combines miniFACE (free-air CO_2_ enrichment) and T-FACE (temperature free-air controlled enhancement) technologies. The miniFACE system (Miglietta et al., 1999) consisted of 2-m diameter PVC rings with laser punctured micro-holes for CO_2_ fumigation into the canopy. At the center of each plot was a CO_2_ transmitter sensor model GMT222 (Vaisala, Helsinki, Finland). A central control unit was responsible for integrating wind speed (obtained at one anemometer located in the center of the Trop-T-FACE facility at 3 m above the ground), and [CO_2_] in the plots. Using a proportional integral derivative (PID) algorithm, the control unit regulated the amount of CO_2_ needed to elevate the [CO_2_] to the set point of 600 μmol mol^-1^ in each plot. A 12-ton cryogenic tank with liquid CO_2_ (-180°C) and a vaporizer unit supplied gaseous CO_2_. Dummy PVC rings without punctures were also placed in plots with ambient [CO_2_]. The daily average [CO_2_] under *aCO*_2_ plots was 412 ± 11 μmol mol^-1^, while FACE fumigation increased diurnal [CO_2_] to 595 ± 20 μmol mol^-1^ under *eCO*_2_ plots during the experiment. Although there was no CO_2_ fumigation during nighttime, due to plant and soil respiration the nighttime average [CO_2_] was 555 ± 19 μmol mol^-1^ in *aCO*_2_ and *eCO*_2_.

The T-FACE system (Kimball et al., 2008) consisted of six infrared Salamander heaters, model TFE 750-240 (Mor Electric Heating, MI, United States) per *eT* plot. The heaters were arranged in a 2-m diameter hexagonal pattern and mounted on Salamander ALEX-F reflectors (Mor Electric Heating, MI, United States) attached to aluminum bars and maintained 0.8 m above the canopy. Heaters warmed the canopy 2°C above the ambient temperature and their height and angle were constantly adjusted with plant growth. Temperature was controlled by a control unit that integrated the canopy temperature (*T_canopy_*) of *aT* and *eT* plots through model SI-1H1-L20 infrared radiometers (Apogee Instruments, UT, United States). Each infrared radiometer was placed in aluminum structures at an angle of 45° in relation to the plant canopy. The voltage to warm each heater was controlled using a PID control system installed in a model CR1000 datalogger with AM25T multiplexors (Campbell Scientific, UT, United States; Kimball et al., 2008). Each warmed plot used a non-warmed plot of the same block as a reference. Dummy heaters were installed at *aT* plots to replicate possible shading over canopy. Through the Loggernet V4 software (Campbell Scientific, UT, United States), we collected and monitored the data of the T-FACE system throughout the experiment.

### Plant Measurements

All analyses were performed in mature expanded leaves developed after clipping and grown under both levels of [CO_2_] and temperature.

#### Leaf and Canopy Temperature

On two different days, the 41st and 54th day of experiment (DOE), we measured the diurnal course of leaf temperature (*T_leaf_*) using a surface infrared thermometer Testo 845 (Testo, Lenzkirch, Germany). Samplings were made in 10 central leaflets per plot every hour. *T_canopy_* of each experimental plot was monitored with the infrared radiometers during the entire experiment and 15 min averages of the canopy temperatures were recorded.

#### Stomatal Density and Stomatal Index

At the end of the experiment, we collected three central leaflets (sun leaves) per plot located at the 5th node from the base to study the effects of the treatments on stomatal distribution at the leaf surface. Sampling was conducted between 13:00 and 14:00 h and leaves were fixed in 50% FAA solution (Johansen, 1941) for 24 h, washed with 50% ethanol for 2 h, and stored in 70% ethanol. Leaf imprints from both leaf surfaces were taken from the central region of the leaf. Samples were digitally photographed (Leica DFC 500, Heidelberg, Germany) using a light microscope (Leica DM4000 B, Heidelberg, Germany). For each sample, we evaluated five different visual fields and counted the number of stomata and epidermal cells. From this, we determined stomatal density per mm^2^ (*SD*) and stomatal index (*SI*) expressed in % (Eq. 1):

$$SI(\%)=\frac{SN}{SN+EC}*100$$

where SN = stomata number and EC = the number of epidermal cells.

The stomatal length (*SL* μm) was measured at the maximum polar length of guard cells for an average of 20 stomata per visual field.

#### Leaf Gas Exchange and Water Status

*In situ* measurements of leaf gas exchange were conducted at three central leaflets (sun leaves) per plot between 9:00 and 11:00 h on the 44th and 66th DOE. Measurements were taken using an LCProSD (ADC BioScientifics, Hoddesdon, United Kingdom). Leaves were kept in the chamber until stomatal conductance and the rate of net photosynthesis stabilized. The measurements were taken at a constant radiation of 1740 μmol m^-2^ s^-1^, temperature of 25°C (in *aCO*_2_*aT* and *eCO*_2_*aT* plots) or 27°C (in *aCO*_2_*eT* and *eCO*_2_*eT* plots), and [CO_2_] of 400 μmol mol^-1^ (in *aCO*_2_*aT* and *aCO*_2_*eT* plots) or [CO_2_] of 600 μmol mol^-1^ (in *eCO*_2_*aT* and *eCO*_2_*eT* plots). We measured the net photosynthesis rate (*A*), stomatal conductance (*g_s_*), transpiration rate (*E*), and calculated the water-use efficiency (*WUE*, *A/E*). Midday water potential (Ψ*_w_*) was measured on the 14, 21, 34, 38, and 55 DOE using a Scholander chamber model 3005 (Soil Moisture Equipment Corp., CA, United States; Scholander et al., 1965). Three branches per plot were cut and sealed in plastic bags containing wet filter paper. Within 1.5 min after cutting, the branches were placed in the pressure chamber for Ψ*_w_* measurements.

#### Chloroplast Ultrastructure

To study the effects of experimental conditions on ultrastructural traits, we collected one central leaflet per plot located at the fourth node from the base. Sampling was conducted at the end of the experiment between 11:00 and 12:00 h. Leaf fragments were cut from the median region of the leaves and fixed in a 2% formaldehyde and 1% glutaraldehyde solution, with a 0.1 M sodium phosphate buffer at pH 7.2 for 24 h, washed in phosphate buffer solution 0.1 M (pH 6.8–7.0; Karnovsky, 1965), and post-fixed in 1% osmium tetroxide for 2 h at 4°C. Leaf segments were dehydrated in an acetone series and embedded in Araldite 6005. Ultrathin cross-sections (c.a. 60 nm) were obtained using an ultramicrotome (Leica Reichert) and contrasted with 2% uranyl acetate for 15 min (Watson, 1958) and lead citrate for 15 min (Reynolds, 1963). Samples were observed using a transmission electron microscope (Philips EM 208).

#### Chlorophyll Fluorescence

After leaf gas exchange measurements were taken on the 66th DOE, the same three sun leaves per plot were collected to measure chlorophyll fluorescence parameters using an imaging-PAM M-series chlorophyll fluorescence system (MINI-version model, Heinz Walz GmbH, Germany; Walz, 2014). Measurements were performed according to Martinez et al. (2014). Leaves were detached, maintained in water, and dark-adapted for 15 min at room temperature to measure dark fluorescence yield (Fo) and the maximum fluorescence yield (Fm). Fo was measured using a low frequency of pulse-modulated measuring light (0.5 μmol m^-2^ s^-1^, 100 μs, 1 Hz), while Fm was measured using a saturation pulse (2700 μmol m^-2^ s^-1^, 0.8 s, 10 Hz). Then, the same leaves were submitted to increasing photosynthetic photon flux density (PPFD) levels (from 0 to 784 μmol photons m^-2^ s^-1^) to measure the effective PS II quantum yield during illumination [Y(II)], the quantum yield of non-regulated energy dissipation [*Y(NO)]*, quantum yield of regulated energy dissipation [Y(*NPQ*)], the “puddle model” coefficient of photochemical quenching (*qP*), the “lake model” coefficient of photochemical quenching of PS II antenna pigment organization (*qL*), and maximum electron transport rate (ETRmax). Images were acquired at maximum PPFD (784 μmol photons m^-2^ s^-1^).

#### Extraction and Determination of Enzymatic Activity

Enzymatic analyses were performed from leaves of different plants collected at midday in each plot at the end of the experimental period. Crude extracts were obtained according to Azevedo et al. (1998), with a few modifications. We macerated 1 g of leaves without the major vein region in liquid nitrogen and solubilized it in 10 mL of 100 mM potassium phosphate buffer (pH 7.5) with 1 mM EDTA, 3 mM DTT, and 2% of PVP (m/v). Crude protein content in each sample was measured at 595 nm according to Bradford (1976).

The superoxide dismutase activity (SOD, EC 1.15.1.1) was measured using the spectrophotometric method as described by Giannopolitis and Ries (1977) and catalase activity (CAT, EC 1.11.1.6) was measured using the spectrophotometric method as described by Azevedo et al. (1998). Ascorbate peroxidase activity (APX, EC 1.11.1.11) was measured using the spectrophotometric method as described by Nakano and Asada (1981). We used the coefficient of molar extinction 3051.4 M^-1^cm^-1^ obtained from our experimental conditions to our calculations. The activities of non-specific peroxidases (POD, EC 1.11.1.7) were measured according to Zeraik et al. (2008) using guaiacol substrate. The results were expressed in μmol min^-1^ mg^-1^ of protein, using the coefficient of molar extinction 26.6 mM^-1^ cm^-1^ (Chance and Maehly, 1955) to our calculations.

#### Photosynthetic Pigments

Leaves from different plants in each experimental plot were used to quantify midday photosynthetic pigments at the end of the experimental period. Measurements were performed according to Lichtenthaler and Wellburn (1983), with a few modifications. We macerated 100 mg of fresh leaves in liquid nitrogen and solubilized samples in 5 mL of 80% acetone. The solution was centrifuged in order to precipitate the biggest particles. From the supernatant, we measured the absorbance at 480, 645, and 663 nm. Thus, we determined the chlorophyll a, chlorophyll b, and carotenoid content of the leaves. From this, we calculated the following proportions: total chlorophyll (a + b) and total chlorophyll/ carotenoid.

#### Lipid Peroxidation

The level of lipid peroxidation of membranes in leaf tissues was measured by the level of malondialdehyde (MDA), which was determined by the thiobarbituric acid (TBA) method, according to Heath and Packer (1968), with a few modifications. We macerated 100 mg of leaves in liquid nitrogen and solubilized in 2.5 mL of 0.1% (m/v) trichloroacetic acid (TCA). An aliquot (500 μL) from the supernatant was transferred to sealed glass tubes, in which 2 mL of 20% TCA with 0.5% TBA were added to the aliquot. This solution was warmed at 95°C for 30 min and then placed on ice to stop the reaction. The supernatant absorbance was determined at 532 and 600 nm. The MDA concentration was calculated using a coefficient of molar extinction of 155 mM^-1^cm^-1^ (Heath and Packer, 1968).

### Data Analysis

For quantitative data, we used a 2 × 2 factorial analysis of variance (ANOVA) to test the main effects of [CO_2_] and temperature levels, as well as their interaction when factors were combined. Data were checked for normality and log-transformed when necessary. We also used the student’s *t*-test to compare means. Analyses were performed using R software 3.2.3, with a significance level of 5% (*p* < 0.05).

## Results

### Meteorology

**FIGURE 1:**
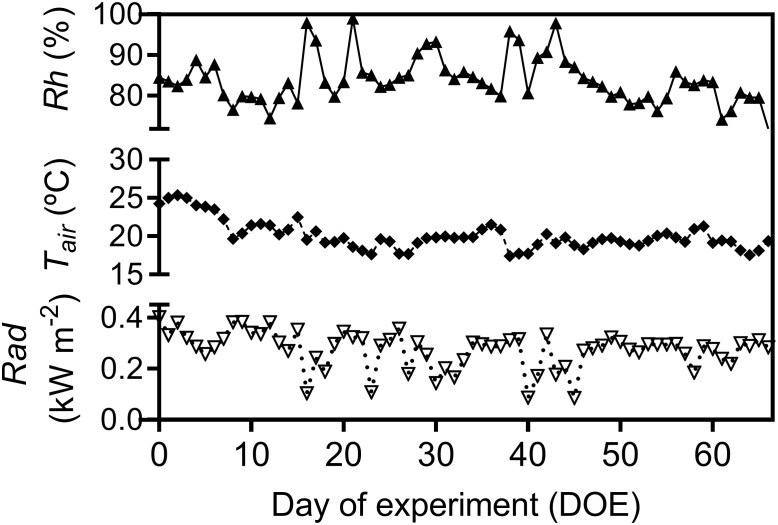
Daily average of relative air humidity (*Rh*), air temperature (*T_air_*) and average diurnal total solar radiation (*Rad*), during the experimental period.

**FIGURE 2:**
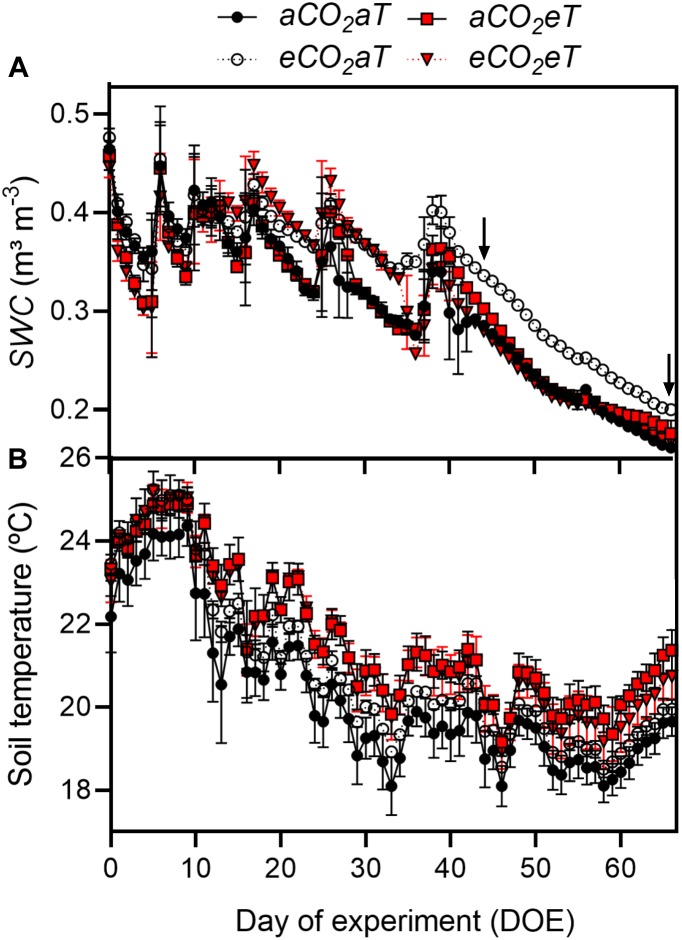
**(A)** Daily average of soil water content (*SWC*) and **(B)** daily average of soil temperature under all treatments during the entire experimental period. Arrows indicate dates in which the leaf gas exchange parameters were measured. Treatments: *aCO*_2_*aT* (ambient [CO_2_] and ambient temperature), *eCO*_2_*aT* (elevated [CO_2_] and ambient temperature), *aCO*_2_*eT* (ambient [CO_2_] and elevated temperature) and *eCO*_2_*eT* (elevated [CO_2_] and elevated temperature). DOE = day of experiment. Stack bars indicate the standard deviation.

Throughout the experiment, air relative humidity (*Rh*), air temperature *(T_air_*), total solar radiation (*Rad*), soil water content (*SWC*), and soil temperature (*T_soil_*) gradually decreased (Figure 1, 2). Days were usually cloud-free with maximum total solar radiation of 0.89 kW m^-2^, and total diurnal average of 0.27 kW m^-2^ (Figure 1). Total precipitation during the experimental period was 120 mm, mostly during the first half of the experimental period. The second half of the experimental period was marked by drier conditions with total rainfall of only 26.7 mm over 41 consecutive days (Supplementary Figure 2B). Average *T_air_* during the experiment was 20.5°C with highest and lowest recorded *T_air_* 32.6 and 7.6°C, respectively. Average *Rh* was 82.2% and the recorded lowest *Rh* during the experimental period was 33% (Figure 1).

### Soil Water Content and Soil Temperature

The first half of the experiment had more frequent rainfall (Supplementary Figure 2B), which caused fluctuations in *SWC* (Figure 2A), whereas in the second half of the experimental period, reduced precipitation caused an almost linear decrease in *SWC* (Figure 2A). At 10 cm deep, *SWC* at *eCO*_2_*aT* remained higher than at *aCO*_2_*aT* plots. During the experiment, average *SWC* at *aCO*_2_*aT* was 0.30 ± 0.07 m^3^ m^-3^ while *eCO*_2_*aT* increased *SWC* to 0.34 ± 0.06 m^3^ m^-3^ (Figure 2A). However, *aCO*_2_*eT* had no effect on average *SWC* (0.30 ± 0.07 m^3^ m^-3^) while *eCO*_2_*eT* counterbalanced the increase in *SWC* promoted by *eCO*_2_*aT* and reduced *SWC* to 0.29 ± 0.07 m^3^ m^-3^. During the entire experiment, soil temperature of *eT* plots was on average 1°C warmer than the *aT* plots (Figure 2B).

### Plant Measurements

#### Canopy and Leaf Temperature

**FIGURE 3:**
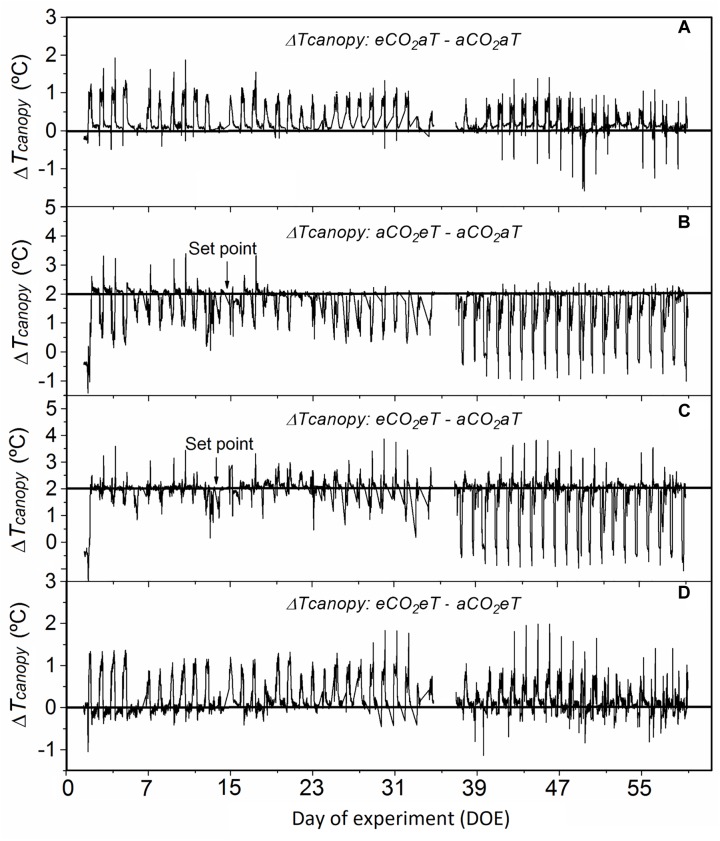
Difference in canopy temperature (Δ *T_canopy_*) of *Stylosanthes capitata* during the experiment. **(A)** Δ *T_canopy_* between *eCO*_2_*aT*–*aCO*_2_*aT* plots. **(B)** Δ *T_canopy_* between *aCO*_2_*eT*–*aCO*_2_*aT* plots. **(C)** Δ *T_canopy_* between *eCO*_2_*eT*–*aCO*_2_*aT* plots. **(D)** Δ *T_canopy_* between *eCO*_2_*eT*–*aCO*_2_*eT* plots. *T_canopy_* was recorded every 15 min by Apogee infrared radiometers. Arrows indicate the point 2°C above the ambient canopy temperature that was set in warmed treatments. The missing values between days 35 and 37 after treatment are due to a dead datalogger battery on these days. Treatments: *aCO*_2_*aT* (ambient [CO_2_] and ambient temperature), *eCO*_2_*aT* (elevated [CO_2_] and ambient temperature), *aCO*_2_*eT* (ambient [CO_2_] and elevated temperature) and *eCO*_2_*eT* (elevated [CO_2_] and elevated temperature).

Compared to *aCO*_2_*aT*, the *eCO*_2_*aT* treatment increased *T_canopy_* during the experimental period (Figure 3A), especially during the hottest hours of the day. The average difference in *T_canopy_* between *eCO*_2_*aT* and *aCO*_2_*aT* during the entire experiment was 0.25°C (Figure 3A). During the experiment, *T_canopy_* of *aCO*_2_*eT* plots was close to the 2°C above ambient setting (*aCO*_2_*aT*), especially during the nighttime. During daytime, *T_canopy_* drifted from the set point as wind and plant transpiration varied. The average difference between *T_canopy_* of *aCO*_2_*eT* and *aCO*_2_*aT* during the entire experiment was 1.6°C (Figure 3B). However, during the experiment, the *eCO*_2_*eT* treatment caused an additive increase in *T_canopy_* from the *eCO*_2_ and *eT* treatments. The average difference in *T_canopy_* between *eCO*_2_*eT* and *aCO*_2_*aT* during the entire experiment was 1.8°C (Figure 3C). The difference in *T_canopy_* between *eCO*_2_*eT* and *aCO*_2_*eT* was approximately 0.2°C (Figure 3D), showing again the synergistic effect of elevated [CO_2_] on the increase in *T_canopy_*.

At *eCO*_2_*aT* daily leaf temperature (*T_leaf_*) increased on average 1.3 and 0.7°C more at 41 and 55 DOE, respectively, than under ambient conditions. The combination of *eCO*_2_ and warming had an additive effect, increasing *T_leaf_* (Supplementary Figure 3).

#### Stomatal Density, Stomatal Index, and Stomatal Size

**FIGURE 4:**
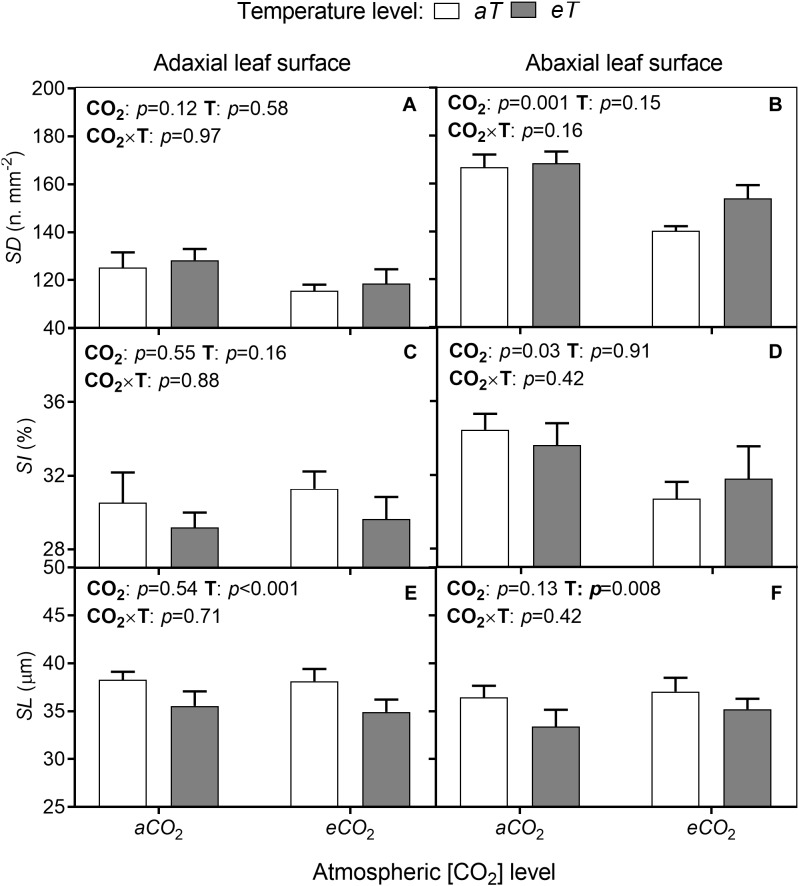
**(A,B)** Stomatal density (*SD*). **(C,D)** Stomatal index (*SI*). **(E,F)** Stomatal length (*SL*) on adaxial leaf surface (left column) and abaxial leaf surface (right column) from leaves of *S. capitata* grown under different levels of [CO_2_] and temperature at 66th day of experiment. [CO_2_] levels: ambient (*aCO*_2_) and elevated (*eCO*_2_, 600 μmol mol^-1^). Temperature levels: ambient (*aT*) and elevated (*eT*, +2°C above ambient canopy temperature). ANOVA *p*-values are shown for main effects (CO_2_ or T) or interactions CO_2_ × T. Stack bars indicate the standard error.

Leaves of *S. capitata* are amphistomatic with higher *SD* on the abaxial surface (lower leaf surface). There was no alteration of this pattern under any experimental condition (Figure 4). There were also no interactions of [CO_2_] × temperature for any stomatal parameters. Furthermore, there were no effects of *eCO*_2_ on *SD* (Figure 4A) or *SI* (Figure 4C) in the adaxial surface (upper leaf surface). However, on the abaxial surface *eCO*_2_ decreased *SD* by approximately 16% (Figure 4B) and *SI* by 11% regardless of temperature level (Figure 4D). Also, *SL* was reduced by *eT* by 7% on average at the adaxial surface (Figure 4E) and 8% at the abaxial leaf surface (Figure 4F), regardless of CO_2_ level.

#### Leaf Gas Exchange and Leaf Water Status

**FIGURE 5:**
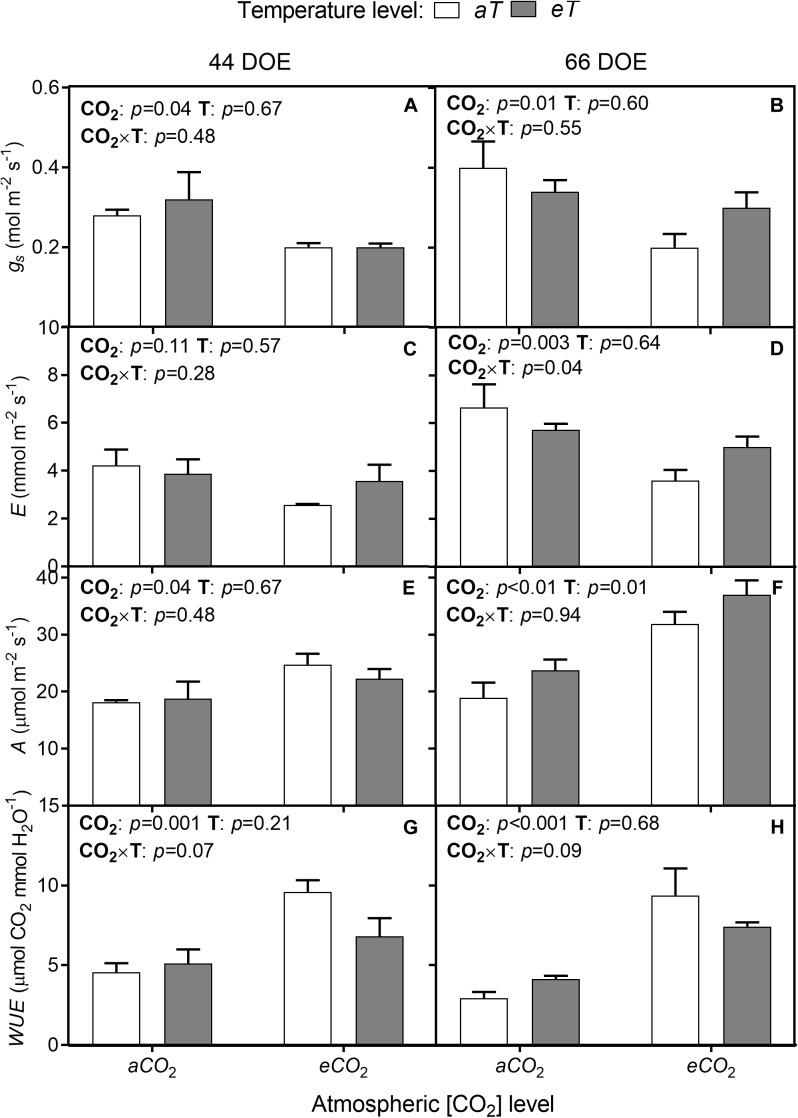
Leaf gas exchange of *S. capitata* performed at 44th (left column) and 66th DOE (right column) under different levels of [CO_2_] and temperature. **(A,B)** Stomatal conductance (*g_s_*). **(C,D)** Transpiration rate *(E)*. **(E,F)** Net photosynthesis rate *(A)*. **(G,H)** Instantaneous water-use efficiency (*WUE*). [CO_2_] levels: ambient (*aCO*_2_) and elevated (*eCO*_2_, 600 μmol mol^-1^). Temperature levels: ambient (*aT*) and elevated (*eT*, +2°C above ambient canopy temperature). DOE = day of experiment. ANOVA *p*-values are shown for main effects (CO_2_ or T) or interactions CO_2_ × T. Stack bars indicate the standard error.

Following the observed reductions in *SD* and *SI*, the *eCO*_2_ treatment resulted in an up to 33% reduction of stomatal conductance (*g_s_*) regardless of temperature level for both sampling days (Figure 5A,B). At 66th DOE, *E* was reduced at *eCO*_2_ treatments (Figure 5C,D), but this response was dependent on the temperature treatment, with warming partially offsetting this reduction. Net photosynthesis rates (*A*) were increased by *eCO*_2_ and by warming (*eT*). At 44th DOE, *A* increased by 27% at *eCO*_2_ regardless of the temperature treatment (Figure 5E). At the end of the experiment, warming also affected *A* by approximately 39% (Figure 5F). At *aCO*_2_*aT* average *A* rates were 18.2 ± 1.7 μmol m^-2^ s^-1^, while rates were 31.2 ± 1.8 and 25.3 ± 2.1 μmol m^-2^ s^-1^ for the *eCO*_2_*aT* and *aCO*_2_*eT* treatments, respectively (Figure 5F). Main effects caused an additive increase without significant interaction for plants grown at *eCO*_2_*eT* (*p <* 0.01, Figure 5F).

The effects of *eCO*_2_ on stomatal aperture, along with increased *A*, improved instantaneous *WUE*. *WUE* increased by approximately 69% and 139% regardless of temperature level at 44th and 66th DOE, respectively (Figure 5G,H). Despite changes in gas exchange, leaf water potential was not modified during the growing season. The lowest values of midday water potential were observed in the second half of the experiment (Table 1), when *SWC* was reduced due to decreased precipitation.

**Table 1:** Midday water potential (MPa) in leaves of *S. capitata* exposed to elevated levels of atmospheric [CO_2_] and temperature at the Trop-T-FACE facility.

*DOE*	*aCO*_2_*aT*	*eCO*_2_*aT*	*aCO*_2_*eT*	*eCO*_2_*eT*	ANOVA *p*-value
14	–0.11 ± 0.01	-0.2 ± 0.05	-0.13 ± 0.02	-0.15 ± 0.04	CO_2_ = 0.15, T = 0.63, CO_2_ × T = 0.39
21	-0.1 ± 0.02	-0.1 ± 0.01	-0.07 ± 0.02	-0.09 ± 0.01	CO_2_ = 0.57, T = 0.35, CO_2_ × T = 0.73
34	-0.18 ± 0.02	-0.21 ± 0.02	-0.24 ± 0.05	-0.22 ± 0.03	CO_2_ = 0.81, T = 0.25, CO_2_ × T = 0.37
38	-0.14 ± 0.02	-0.11 ± 0.03	-0.13 ± 0.01	-0.12 ± 0.03	CO_2_ = 0.58, T = 0.94, CO_2_ × T = 0.67
55	-0.42 ± 0.20	-0.37 ± 0.06	-0.59 ± 0.10	-0.47 ± 0.10	CO_2_ = 0.33, T = 0.12, CO_2_ × T = 0.65


*Mean values (*n* = 4) and standard error (±) are show for every sampling day. [CO_*2*_] levels: ambient (*aCO_*2*_*) and elevated (*eCO*_2_, 600 μmol mol^-*1*^). Temperature levels: ambient (*aT*) and elevated (*eT*, +2°C above ambient canopy temperature). ANOVA *p*-value is shown for main effects (CO_*2*_ or T) or interactions CO_*2*_ × T. DOE: day of experiment.*

#### Chloroplasts Ultrastructural Qualitative Analysis

**FIGURE 6:**
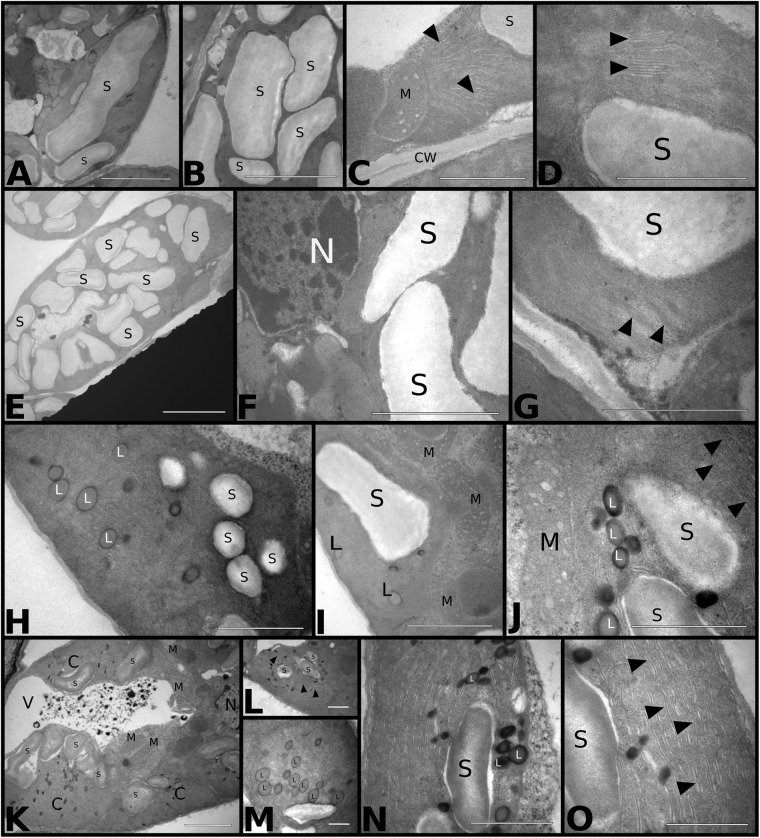
Transmission electron micrograph of *S. capitata* leaves grown under different levels of [CO_2_] and temperature at 66th day of experiment. **(A–D)** *aCO*_2_*aT* samples. **(A,B)** Longitudinal section of chloroplast showing large and numerous starch grains. Scale bar of 10 μm in panels **(A,B)**. **(C)** Longitudinal section of the terminal portion of a chloroplast associated with mitochondria. Arrowheads show intact thylakoid membranes. Scale bar 3 μm. **(D)** Details of thylakoid membranes of an intact chloroplast (arrowhead). Scale bar 2 μm. **(E–G)** *eCO*_2_*aT* samples. **(E)** Overview of a chloroplast in longitudinal section showing a large number of starch grains. Scale bar 10 μm. **(F)** Enlarged cell. Scale bar 4 μm. **(G)** Details of a chloroplast showing the integrity of thylakoid membranes (arrowhead). Scale bar 3 μm. **(H–J)** *aCO*_2_*eT* samples **(H)** Overview of a chloroplast from leaf mesophyll with a reduced number and size of starch grains and the presence of plastoglobuli in the stroma of chloroplast. Scale bar 3 μm. **(I,J)** Detail of chloroplasts with a greater association with mitochondria. **(J)** Evidence that the internal membranes are less organized in the thylakoid (arrowhead). Scale bars 3 μm in **(I)** and 2 μm in **(J)**. **(K–O)** *eCO*_2_*eT* samples. **(K)** Overview of the mesophyll cell with chloroplasts in association with mitochondria and vacuole with presence of electrodense amorphous material. Scale bar 5 μm. **(L)** Detail of a chloroplast in longitudinal section. Arrowhead – internal membranes of chloroplasts. Scale bars 2 μm. **(M)** Detail of chloroplasts in cross section showing the large number of plastoglobuli in the stroma. Scale bar 2 μm. **(N,O)** Details of chloroplasts showing plastoglobuli, starch grains, and internal membranes not associated in thylakoids (arrowhead). Scale bar 3 μm in N and O. Abbreviations: S, starch grain; M, mitochondria; CW, cell wall; N, nucleus; L, plastoglobuli; C, chloroplasts; V, vacuole.

We used transmission electron microscopy (TEM) to study the effects of temperature and CO_2_ treatments on the chloroplast ultrastructure of palisade mesophyll cells (Figure 6). Plants grown at *aCO*_2_*aT* had well-developed chloroplasts with organized thylakoid membranes and large starch grains (Figure 6A,B,D). Few cells exhibited the typical association between chloroplasts and mitochondria related to photorespiration (Figure 6C). Plants grown in the *eCO*_2_*aT* treatment showed an increase in the size and number of chloroplasts as well as the size of starch grains in palisade mesophyll cells when compared to control plants (Figure 6E,F) with no changes in thylakoid membrane structure (Figure 6G). In the *aCO*_2_*eT* treatment, plants showed a reduction in the size and number of chloroplasts and starch grains but an increase in the number and size of plastoglobuli in their stroma and an increase in the spacing of thylakoid membranes when compared to *aCO*_2_*aT* (Figure 6H–J). There was a tighter association between chloroplasts and mitochondria in *aCO*_2_*eT* plots (Figure 6I,J) than in controls. Plants grown at *eCO*_2_*eT* exhibited a reduction in the number and size of starch grains (Figure 6K–O), an increase in the number of stroma plastoglobuli (Figure 6M,N) and had poor stacking of thylakoid membranes (Figure 6L,O) when compared to controls. These plants also had a tight association between chloroplasts and mitochondria (Figure 6K).

#### Chlorophyll Fluorescence

**FIGURE 7:**
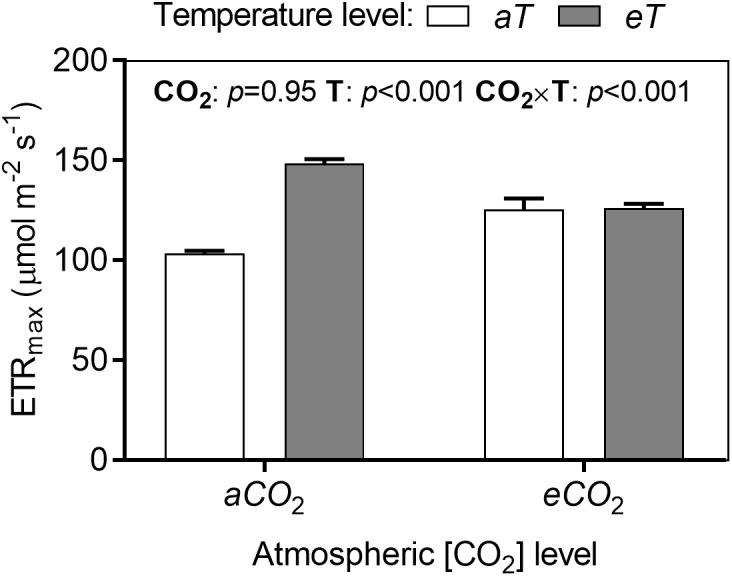
Maximum electron transport rate (ETR*_max_*) of *S. capitata* leaves under different levels of atmospheric [CO_2_] and temperature at 66th day of experiment at maximum PPFD (784 μmol photons m^-2^ s^-1^). [CO_2_] levels: ambient (*aCO*_2_) and elevated (*eCO*_2_, 600 μmol mol^-1^). Temperature levels: ambient (*aT*) and elevated (*eT*, +2°C above ambient canopy temperature). ANOVA *p*-values are shown for main effects (CO_2_ or T) or interactions CO_2_ × T. Stack bars indicate the standard error.

The analysis of the rapid light-induced fluorescence curves showed significant effects of treatments for many fluorescence parameters during increasing stages of PPFD. We compared the treatments in each parameter at maximum irradiance (784 μmol photons m^-2^ s^-1^). The parameter ETR*_max_* increased in warmed plots depending on CO_2_ level by approximately 22% (Figure 7).

**FIGURE 8:**
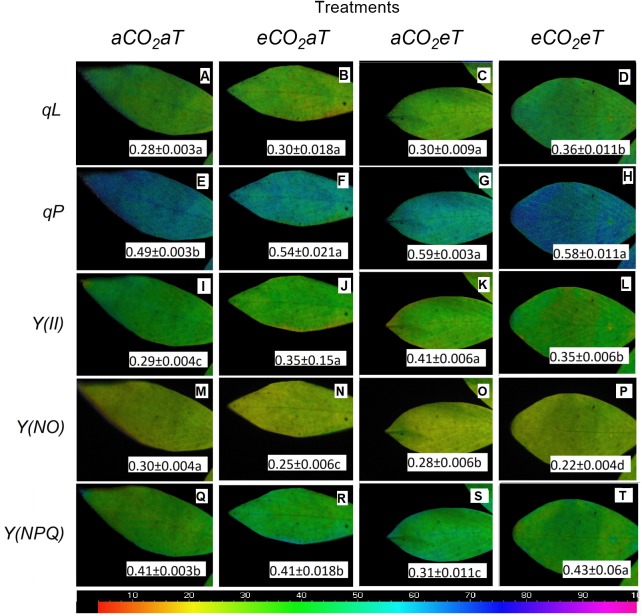
Images of chlorophyll fluorescence parameters made in leaves of *S. capitata* exposed to different levels of atmospheric [CO_2_] and temperature at maximum PPFD (784 μmol photons m^-2^ s^-1^). **(A–D)** Coefficient of photochemical quenching (lake model) *(qL)*. **(E–H)** Coefficient of photochemical quenching (puddle model) (*qP*). **(I–L)** Effective PSII quantum yield Y(II). **(M–P)** Quantum yield of non-regulated energy dissipation Y(NO). **(Q–T)** Quantum yield of regulated energy dissipation Y(NPQ). Measurement was performed at 66th day of experiment. [CO_2_] levels: ambient (*aCO*_2_) and elevated (*eCO*_2_, 600 μmol mol^-1^). Temperature levels: ambient (*aT*) and elevated (*eT*, +2°C above ambient canopy temperature). Different letters in the same row indicate statistical differences between treatments determined by *t*-test. Relative values of each parameter in the images have been mapped to a color palette ranging from 0 to 1 and displayed using an identical false color scale (bar is at the bottom of image).

The coefficient of photochemical quenching (lake model) (*qL*) (Figure 8A–D), the coefficient of photochemical quenching (puddle model) (*qP*) (Figure 8E–H), the effective PSII quantum yield Y(II) (Figure 8I–L), and the quantum yield of regulated energy dissipation [Y(NPQ); Figure 8Q–T] increased under warmed plots depending on [CO_2_] level by 28, 18, 20, and 5%, respectively. The ANOVA analysis showed that Y(NO) decreased due to the main effects of *eCO*_2_ and *eT* by approximately 17% (Figure 8N) and 7% (Figure 8O), respectively. For the *eCO*_2_*eT* treatment, Y(NO) decreased by 27% (Figure 8P) compared to the *aCO*_2_*aT* (student’s *t*-test comparison). This same phenomenon was seen for *qL*, where isolated effects of *eCO*_2_ and *eT* increased *qL* by 7% (Figure 8B,C), while under the combination *eCO*_2_*eT* a synergistic effect improved *qL* by 28% (Figure 8D) when compared to *aCO*_2_*aT* (student’s *t*-test comparison).

#### Enzymatic Activity, Pigments and Malondialdehyde Levels

The activity of catalase (CAT), peroxidase (POD), superoxide dismutase (SOD), and malondialdehyde levels (MDA) were not affected by any treatment. However, ascorbate peroxidase (APX) activity decreased under *eT* regardless of the [CO_2_]. Total chlorophyll (chlorophyll a + b) was not changed by treatments; however, carotenoid content increased under *eCO*_2_*eT* by about 36% and the chlorophyll-to-carotenoid ratio increased at *eT* regardless of the [CO_2_] level at which plants were grown (Table 2).

**Table 2:** Biochemical parameters in leaves of *S. capitata* exposed to elevated levels of atmospheric [CO_2_] and temperature at the Trop-T-FACE facility at 66th day of experiment.

Parameter	*aCO*_2_*aT*	*eCO*_2_*aT*	*aCO*_2_*eT*	*eCO*_2_*eT*	ANOVA *p*-values
CAT	161.7 ± 10	139.4 ± 16.3	156.7 ± 6.8	143.3 ± 6.7	CO_2_ = 0.12, T = 0.95, CO_2_ × T = 0.68
APX	0.10 ± 0.01	0.15 ± 0.02	0.07 ± 0.02	0.07 ± 0.01	CO_2_ = 0.051, T = 0.001, CO_2_ × T = 0.08
POD	0.10 ± 0.02	0.14 ± 0.04	0.07 ± 0.01	0.08 ± 0.01	CO_2_ = 0.21, T = 0.06, CO_2_ × T = 0.33
SOD	21.8 ± 1.0	23.3 ± 0.7	19.8 ± 2.2	20.6 ± 0.6	CO_2_ = 0.39, T = 0.09, CO_2_ × T = 0.78
MDA	177.4 ± 7.8	153.8 ± 28.2	175.1 ± 41.5	219.0 ± 40.1	CO_2_ = 0.63, T = 0.77, CO_2_ × T = 0.65
Chl	5.7 ± 1.00	5.0 ± 0.6	6.3 ± 0.6	8.8 ± 1.5	CO_2_ = 0.38, T = 0.051, CO_2_ × T = 0.13
Carot	2.3 ± 0.30	1.8 ± 0.2	2.4 ± 0.1	3.2 ± 0.3	CO_2_ = 0.55, T = 0.01, CO_2_ × T = 0.02
Chl/Carot	2.4 ± 0.05	2.6 ± 0.07	2.8 ± 0.04	2.7 ± 0.9	CO_2_ = 0.31, T = 0.002, CO_2_ × T = 0.06


*Mean values (*n* = 4) and ±standard errors are show for catalase (CAT), ascorbate peroxidase (APX), peroxidase (POD), superoxide dismutase (SOD), malondialdehyde (MDA), total chlorophyll (Chl), and carotenoid (Carot). All variables are expressed in μg gDW^-*1*^. *aCO_*2*_aT* = ambient [CO_*2*_] and ambient temperature; *eCO_*2*_aT* = elevated [CO_*2*_] and ambient temperature; *aCO_*2*_eT* = ambient [CO_*2*_] and elevated temperature; *eCO_*2*_eT* = elevated [CO_*2*_] and elevated temperature. ANOVA *p*-value is shown for main effects (CO_*2*_ or T) or interactions CO_*2*_ × T.*

## Discussion

In this study, we explored the interacting effects of two opposing abiotic forcing factors (temperature and CO_2_) on stomatal regulation of a tropical forage species grown in the field. Our key findings were: (i) *eCO*_2_ had an influence on *g_s_*, SD, and SI; (ii) *eT* reduced the size of stomata; (iii) *eT* partially or fully mitigated the effects *eCO*_2_ effects on *E* and *SWC*; and (iv) *eCO*_2_ augmented the increase in leaf temperature over that of the experimental warming treatment. These results suggest plant photosynthetic performance and water use efficiency may be enhanced in response to the combination of high [CO_2_] and temperature for this tropical foraging species.

As expected, *g_s_* was reduced by *eCO*_2_ during the growing season by 33% on average in both temperature treatments, similar to the reduction reported in a meta-analysis by Ainsworth and Rogers (2007) who reported an average *g_s_* reduction 22% across all plant species in FACE studies. Reduced *g_s_* under *eCO*_2_ is consistent in long-term experiments, with no strong indication of reduced sensitivity (Herrick et al., 2004; Marchi et al., 2004; Leakey et al., 2006). The number of studies reporting the combined effect of *eCO*_2_ and warming on *g_s_* is very limited, and the existing studies report little or no interaction between *eCO*_2_ and *eT* (Naudts et al., 2013; Fauset et al., 2018). This means that the effects of these abiotic forcing factors are generally independent. Our findings are supported by these studies, as we also found no effect of interaction between [CO_2_] and temperature levels. We also found similar magnitude of effects of *eCO*_2_ on *g_s_* regardless of the temperature level and across different soil moisture conditions (0.35 m^3^ m^-3^ and 0.15 m^3^ m^-3^ in the first and second gas exchange sampling, respectively). This is presumably associated with the high drought tolerance of *S. capitata* (Bill, 1992), which develops deep roots for water uptake in deep soil profile layers (Williams and Gardener, 1984). A closely related species such as *S. hamata* produces roots up to 70 cm long even in compacted soils (Lesturgez et al., 2004). It is important to note that *SWC* data were measured between 0 and 10 cm deep and may not reflect full soil profile conditions. Therefore, even under restricted superficial soil moisture, *g_s_* and gas exchange can still be sustained by deeper soil layers moisture (Kirschbaum and McMillan, 2018).

Other important effects of *eCO*_2_ were found in our study such as the coinciding decrease in *SD* and *SI* which co-vary with the reduced *g_s_*, indicate that elevated [CO_2_] affected stomata differentiation on the abaxial leaf surface of this species. These changes in *SD* and *SI* optimize gas exchange for greater carbon fixation while providing better control over water loss via transpiration (Lin et al., 2001; Nadeau and Sack, 2002; Franks et al., 2012; Pillitteri and Torii, 2012). The decreases in stomatal number under *eCO*_2_ are also reported for both leaf surfaces in other species. For example, Teng et al. (2006) found that leaves of *Arabidopsis thaliana* (Brassicaceae) grown under 700 μmol mol^-1^ of [CO_2_] had *SD* reduced by 19% and 14% at adaxial and abaxial leaf surfaces, respectively, with a consequent decrease of *g_s_*. Therefore, *g_s_* was presumably reduced owing to the combination of decreased stomatal aperture and stomatal numbers. This same result was found by Habermann et al. (2019b) in leaves of the C_4_ tropical forage species *Panicum maximum* under *eCO*_2_ combined with warming.

Direct effects of warming over *g_s_* are not always simple to measure because other variables such as leaf water status and *VPD* co-vary with air temperature (Urban et al., 2017). Isohydric leaves adjust their *g_s_* in response to leaf water potential and *VPD* (Klein, 2014). However, there were no effects of *eT* on water status of plants during the entire experiment, which partially explains the absence of *g_s_* responses to warming. Despite infrared warming in the T-FACE system often increasing *VPD* in warmed plots (Kimball, 2005), the *VPD* increment observed in our experiment did not affect *g_s_*, presumably because this species is adapted to warm and arid environments with leaf trichomes, presumably affecting microclimate and acting as a buffer to moderate *VPD* increases. Regardless of [CO_2_] level, plants grown under *eT* had smaller stomata on both leaf surfaces, which might be an adaptation that allows plants to keep the stomata open under water stress conditions (Spence et al., 1986) that frequently accompany heat in tropical regions. This is because smaller stomata increase resistance to gas diffusion (Drake et al., 2013) limiting water loss whilst still allowing carbon uptake under prolonged drought (Spence et al., 1986). Smaller stomata have also been suggested to exhibit a faster response to environmental changes than larger stomata due to a larger surface area to volume ratio in guard cell membranes, allowing faster solute influx or efflux (Drake et al., 2013; Raven, 2014). However, the relationship between stomatal size and kinetics may not be conserved over a wide range of species and possibly will only occur in species with comparable stomata structures (Lawson and Vialet-Chabrand, 2019). Our study found no evidence of an opposing interaction force of temperature against *eCO*_2_ and its effects on *g_s_* and anatomical changes. Both main effects produced leaves with fewer and smaller stomata under *eCO*_2_ and may have improved C uptake and water use simultaneously.

Water-saving mechanisms observed under *eCO*_2_*aT* were supported by the *SWC* data where soil moisture under ambient temperature remained higher during the experiment, presumably because of less soil water uptake by roots. Soil moisture maintenance can benefit plants during water shortages periods (Leakey et al., 2009; AbdElgawad et al., 2015) because plants will sustain photosynthesis for longer under these conditions (Allen et al., 2011). However, under *eCO*_2_*eT*, *SWC* was not conserved, probably owing to the greater *VPD* and soil evapotranspiration caused by warming. The increase in *WUE* indicates an enhancement of carbon (C) fixation per unit of water transpired in changing conditions. Improved *WUE* was also observed in sugarcane (*Saccharum officinarum*, Poaceae; Marin et al., 2013) and coffee (*Coffea arabica*, Rubiaceae; Ghini et al., 2015) developed under *eCO*_2_. A greater *WUE* is also related to enhanced drought tolerance (Mega et al., 2019), which could enable *S. capitata* plants to better tolerate dry environments and improve the resistance under seasonal water shortages (Wullschleger et al., 2002). Studies performed with other C_3_ grasses (Naudts et al., 2013) and legumes (AbdElgawad et al., 2015) have shown that elevated CO_2_ has the potential to compensate the impacts of drought and warming on plant photosynthesis and growth. This response would be especially important in the tropics, as climate models predict a significant reduction in rainfall and increased incidences of extreme drought in the next few decades (Lyra et al., 2017). In our study, *eCO*_2_ improved *WUE* in very different conditions of soil moisture and ambient temperature levels, indicating that *S. capitata* has the potential to adjust to future conditions of temperature and soil water availability. Very few studies have combined high [CO_2_], warming, and drought together (Dias de Oliveira et al., 2013), and in wheat (*Triticum aestivum*, Poaceae) interactions between all three variables on *WUE*, photosynthesis, and biomass were not found (Dias de Oliveira et al., 2015a,b). However, further studies are necessary to better understand the interactions between CO_2_ × drought × warming on plants’ water use and how different physiological parameters co-vary within climatic variation.

In soybeans (*Glycine max*, Fabaceae) grown in a temperate climate, for every 10% reduction in *g_s_* a decrease of 8.6% in evapotranspiration is expected (Bernacchi et al., 2007). However, decreases in leaf-level *g_s_* should be carefully extrapolated to the ecosystem level because many negative feedbacks can offset or even reverse this straight-forward response (Marchi et al., 2004). Here, we observed that warming partially offset the reduction of *E* by *eCO*_2_, which is presumably associated with increased *VPD* in warmed plots. According to calculations performed on other plant species, a 10% decrease in *g_s_* under *eCO*_2_ would be enough to mitigate an increase in *g_s_* caused by a warming of 1°C in air temperature (Kirschbaum and McMillan, 2018).

Interestingly, warming alone did not increase *E* during the experiment. One possible explanation is that leaf temperature (*T_leaf_*) was higher under *eCO*_2_*eT* than under *aCO*_2_*eT*, due to the additive effects of heating caused by the T-FACE warming system and elevated [CO_2_]-induced stomatal closure. In canopies with less turbulence, such as grasslands, stomatal closure increases *T_leaf_* (Kirschbaum and McMillan, 2018). Here, *T_leaf_* increased on average 2.6 and 1.8°C, under *eCO*_2_*eT* and *aCO*_2_*eT*, respectively, when compared with *aCaT*. Leaf temperature represents a balance between the net heat gain from short- and long-wave radiation, heat loss by heat exchange (convection and conduction), latent heat loss by evapotranspiration, heat stored in metabolic processes, and physical heat storage (Jones and Rotenburg, 2011). The effects of *eCO*_2_ in *T_leaf_* were more intense in moments of increased solar radiation but were not significant in the early and late hours of the day. The changes in *T_leaf_* were coupled with total incoming solar radiation, which indicates that the leaf boundary layer was thin, probably due to the small size of the leaves (Jones and Rotenburg, 2011).

Increases in *T_leaf_* is beneficial when plants are below their optimum temperature of photosynthesis and increases biomass production (Ghannoum et al., 2000). Martinez et al. (2014) found that moderate canopy warming (+2°C) increased leaf area index by 32% and biomass by 16% in *S. capitata*. They also observed a significant increase in the electron transport rate (ETR) and the effective PSII quantum yield Y(II) in leaves under warming compared to leaves at *aT*. In grassland communities composed by grasses, forbs, and legumes, Bloor et al. (2010) found that a 3.2°C warming increased carbon uptake and biomass regardless of the [CO_2_] or drought conditions. Increased rates of *A* (like those observed under *eCO*_2_ and *eT*) may not always be translated into more biomass for foraging as in many cases there is increased allocation to roots and increased respiration from specific tissues (Gonzalez-Meler et al., 2004). We have to point out that photosynthesis measurements were made matching each growing condition and not all measurements were made under the same condition. We wanted to measure point values of net photosynthesis to reflect what plants were experiencing, not a measurement to compare photosynthetic potentials or photosynthesis acclimation. Because *S. capitata* is a perennial species, measurements of acclimation could be informative; however, it was a short-term experiment and the dynamics of its response to the environment were preferred here. In our study, the main effects of *eCO*_2_ and *eT* greatly increased *A*, and under *eCO*_2_*aT*, *A* increase was probably associated with increased intercellular CO_2_ concentration (*C_i_*), while under *aCO*_2_*eT* and *eCO*_2_*eT*, *A* improvement was probably associated with enhanced ETR. It makes intuitive sense that plants grown under *eT* would have improved *A* since the products of linear electron transport, ATP and NADPH, are used in downstream processes in photosynthetic carbon assimilation (the Calvin cycle; Murchie and Lawson, 2013). We observed that *eCO*_2_*aT* and *eCO*_2_*eT* reduced the quantum yield of non-light-induced non-photochemical fluorescence quenching [Y (NO)] (Figure 8N,O). This reduction in quantum yield was probably caused by a greater fraction of closed PSII centers, which led to a significant increase in the Y(II) value compared to *aCO*_2_*aT*. On the other hand, Y(*NPQ*) was not altered by *eCO*_2_*aT* (Figure 8R) when compared to *aCO*_2_*aT* (Figure 8Q), suggesting that elevated CO_2_ improved *A* and served as the main sink for ATP and NADPH, potentially lowering the requirement for thermal dissipation of energy (Pan et al., 2018).

The combination *eCO*_2_*aT* increased *A* which resulted in an increase in the number and size of starch grains in the chloroplasts of photosynthetic cells, as observed in Figure 6E,F. According to the MDA levels and intact ultrastructure of thylakoid membranes of chloroplasts under this treatment, we observed that elevated [CO_2_] greatly affected stomatal functioning and anatomy, benefiting the photosynthetic performance and *WUE*. We also observed that *eT* significantly reduced the size and number of starch grains regardless of [CO_2_] (Figure 6). Starch is the primary storage carbohydrate in plants and plays an essential role in the response of plants to abiotic stress. It is mobilized as sucrose and transported through the plant in complex source-sinks networks. When the sink is strong, a source tissue has a decreased starch pool because starch is exported. Presumably, warming is driving starch breakdown and transportation. This same stimulus of starch remobilization was found in the tropical forage species *Panicum maximum* under 2°C warming in the T-FACE system (Habermann et al., 2019a,b). In addition, warming promoted a tighter association between chloroplasts and mitochondria (Figure 6I–K), suggesting a mechanism known as photorespiratory CO_2_ scavenging, which increases the likelihood of CO_2_ being re-fixed by chloroplasts (Betti et al., 2016). We also observed fewer membranes organized in a thylakoid structure, but no negative effects on photosynthetic performance were detected.

Legume species have been poorly represented at the biochemical level in climate change studies (AbdElgawad et al., 2015). Here, antioxidant defenses were not affected, except by decreased APX activity under *eT* regardless of [CO_2_] level, which may indicate that ROS is presumably being scavenged. This is because ROS content is one of the stimuli of antioxidant enzyme production and activity (Tripathy and Oelmüller, 2012; AbdElgawad et al., 2015). It has been suggested that *eCO*_2_ can ameliorate drought and warming’s impacts on antioxidative defenses (AbdElgawad et al., 2015). In this study, the enhanced content of carotenoids under *eCO*_2_*eT* indicated improved heat dissipation, which is in agreement with our Chlorophyll Fluorescence data. In addition, the number and size of plastoglobuli greatly increased under *aCO*_2_*eT* and *eCO*_2_*eT* treatments (Figure 6). Plastoglobuli are lipoprotein structures surrounded by a monolayer membrane that is attached to thylakoid membranes of chloroplasts, and its occurrence often increases under abiotic stress under abiotic stress (Austin et al., 2006). Several substances were identified as part of the chemical composition of plastoglobuli, such as triacylglycerol, α-tocopherol, plastoquinol-9, and carotenoids that mostly belong to the xanthophyll cycle, such as xanthophylls zeaxanthin, antheraxanthin, and violaxanthin. Many of these substances act as ROS scavengers and can improve heat dissipation in plants (van Wijk and Kessler, 2017). Thus, the increase in the plastoglobuli is presumably driving plant acclimation to warmer conditions.

In conclusion, our results indicate that elevated [CO_2_] was the main driver of the observed changes in stomatal opening control and anatomy, while warming showed more pronounced effects on PSII performance and antioxidant defenses. The physiological performance of *S. capitata* under the combination *eCO*_2_*eT* indicates great resilience to challenging conditions such as drought. These findings show how important *S. capitata* can be for maintaining grassland productivity under the predicted climate change conditions.

## Author Contributions

EH and EDdO wrote the manuscript. EH, DC, CAM, EDdO, JABSM, and LC collected data in the field and processed the data. CAM and MAG-M conceived and designed the experiments. All authors contributed to the revision of the manuscript.

## Conflict of Interest Statement

The authors declare that the research was conducted in the absence of any commercial or financial relationships that could be construed as a potential conflict of interest.
