# Supplementary material for: Stomatal Development and Conductance of a Tropical Forage Legume Are Regulated by Elevated [CO2] Under Moderate Warming
Source: Front Plant Sci. 2019 May 31;10:609. doi: 10.3389/fpls.2019.00609 (PMC6554438; doi:10.3389/fpls.2019.00609)
Supplement: Supplementary file 1 [file Image_1.pdf]

Supplementary Material

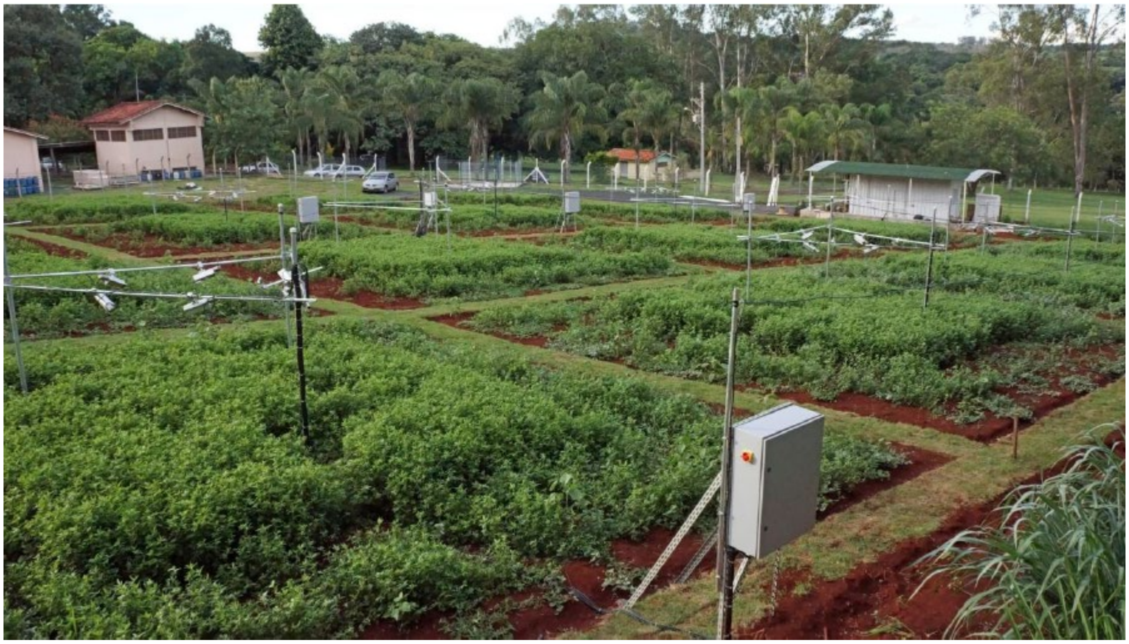

Supplementary Figure 1. Trop-T-FACE facility during *S. capitata* establishment.
