# Supplementary material for: Stomatal Development and Conductance of a Tropical Forage Legume Are Regulated by Elevated [CO2] Under Moderate Warming
Source: Front Plant Sci. 2019 May 31;10:609. doi: 10.3389/fpls.2019.00609 (PMC6554438; doi:10.3389/fpls.2019.00609)
Supplement: Supplementary file 2 [file Image_2.pdf]

Supplementary Material

Supplementary Material

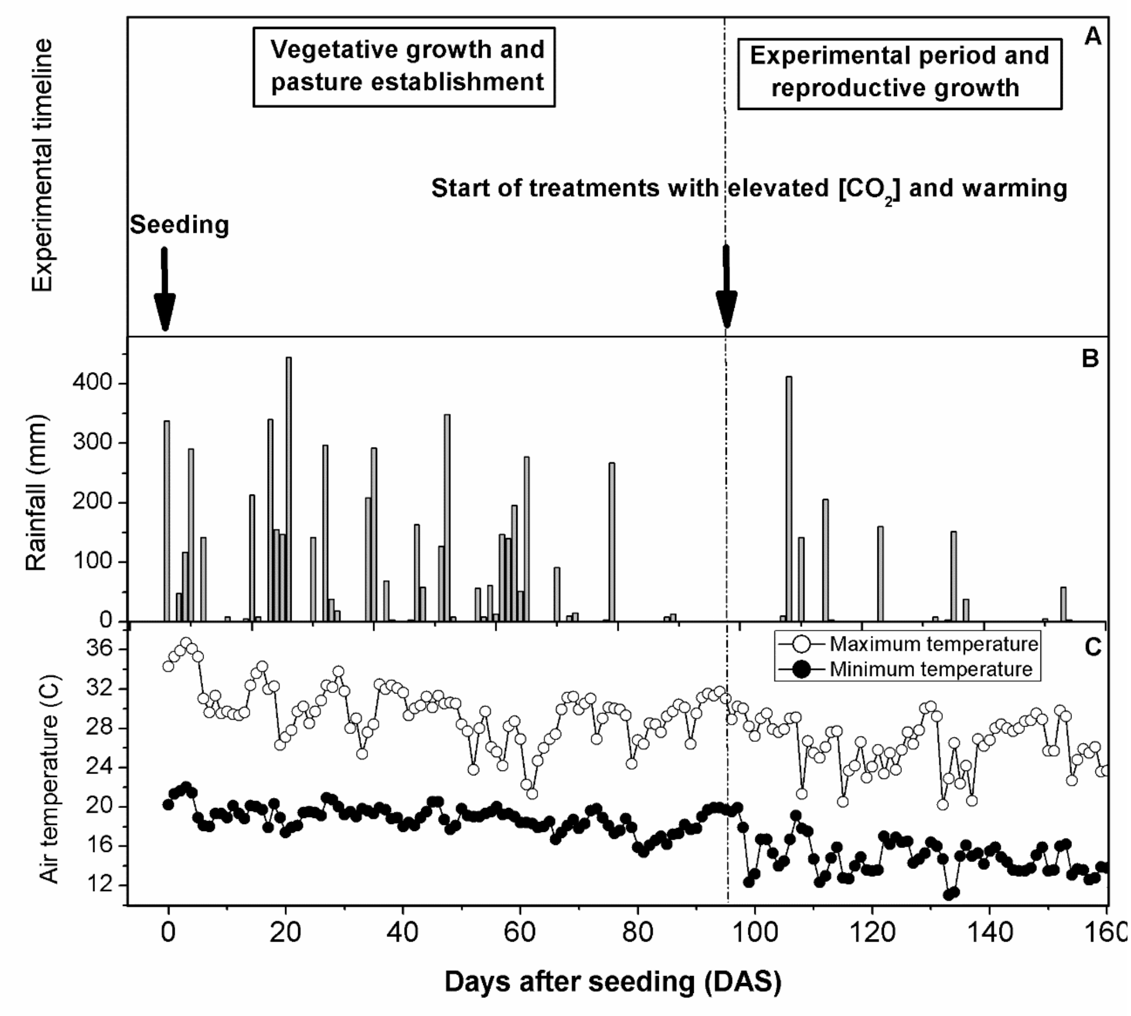

Supplementary Figure 2. Experimental timeline at Trop-T-FACE facility. During seedling growth (A), intense rainfall (B) and high temperatures (C) stimulated plant growth. Experimental conditions were applied only after pasture establishment, which occurred approximately 3 months after the seeding (DAS). To maintain uniform plant height, at 100 DAS we clipped plants 30 cm aboveground and immediately started the treatments with elevated [CO_2] and warming. During regrowth, plants reached anthesis and experiment was conducted during plant reproductive stage. Dashed line separates the experimental period (right side of line) and the establishment of pasture before treatments (left side of the line). DAS = days after seeding.
