# Supplementary material for: Stomatal Development and Conductance of a Tropical Forage Legume Are Regulated by Elevated [CO2] Under Moderate Warming
Source: Front Plant Sci. 2019 May 31;10:609. doi: 10.3389/fpls.2019.00609 (PMC6554438; doi:10.3389/fpls.2019.00609)
Supplement: Supplementary file 3 [file Image_3.pdf]

Supplementary Material

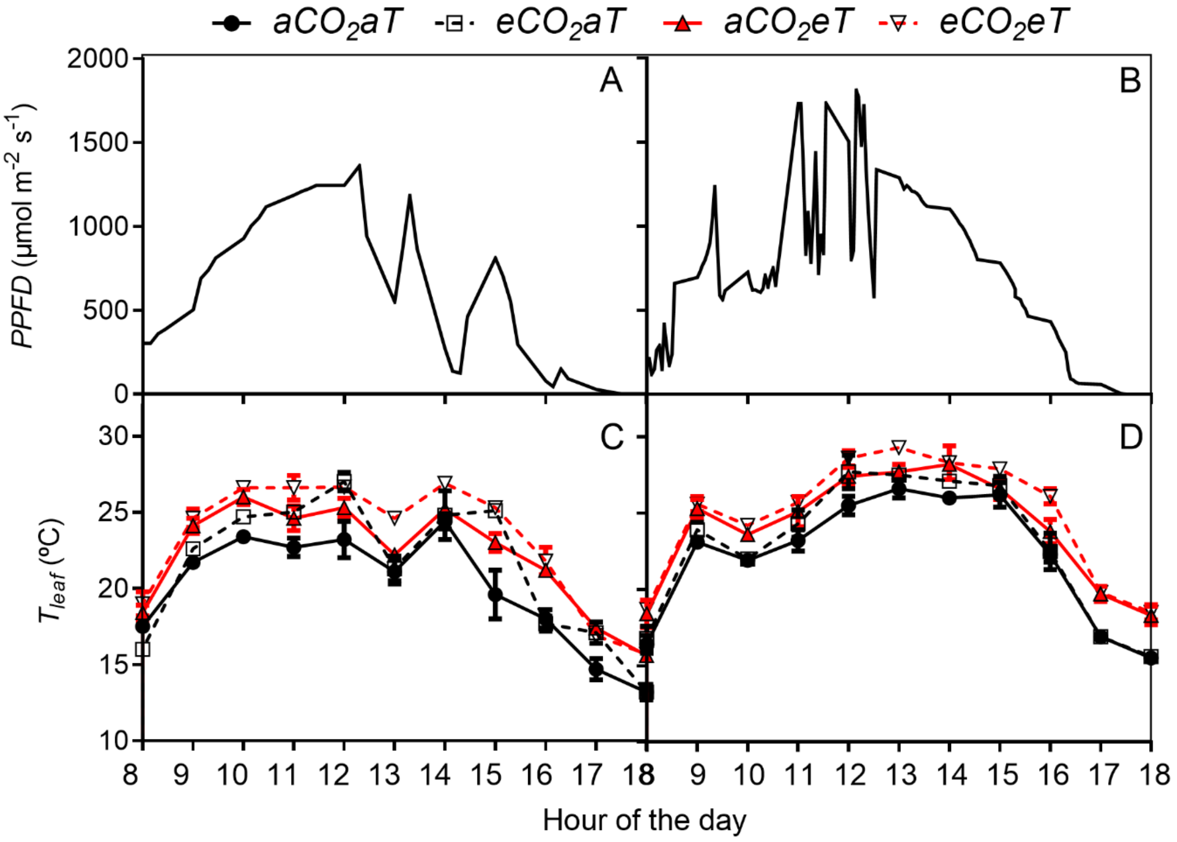

Supplementary Figure 3. Diurnal courses of leaf temperature (*T_{leaf}*) measured directly on leaves of *Stylosanthes capitata* during two days. Left column (A, C) show diurnal course measured at 41st DOE, while right column (B, D) shows diurnal course at 54th DOE. PFFD = Photosynthetically active radiation. Treatments: *aCO_2aT* (ambient [CO_2] and ambient temperature), *eCO_2aT* (elevated [CO_2] and ambient temperature), *aCO_2eT* (ambient [CO_2] and elevated temperature) and *eCO_2eT* (elevated [CO_2] and elevated temperature). Stack bars indicate the standard error.
